# Successful conversion therapy for unresectable hepatocellular carcinoma is getting closer: A systematic review and meta-analysis

**DOI:** 10.3389/fonc.2022.978823

**Published:** 2022-09-13

**Authors:** Yinxuan Pei, Weiwei Li, Zixiang Wang, Jinlong Liu

**Affiliations:** Department of Hepatobiliary Surgery, Affiliated Hospital of Chengde Medical University, Chengde, China

**Keywords:** hepatocellular carcinoma, conversion therapy, chemotherapy, transcatheter arterial chemoembolization, targeted therapy, immunotherapy, combined locoregional-systemic therapy, meta-analysis

## Abstract

**Background:**

Conversion therapy provides selected patients with unresectable hepatocellular carcinoma the opportunity to undergo a curative hepatectomy and achieve long-term survival. Although various regimens have been used for conversion therapy, their conversion rate and safety remain uncertain. Therefore, we conducted some meta-analyses to evaluate the efficacy and safety of several conversion regimens in order to elucidate the optimal regimen.

**Method:**

We performed systematic literature research on PubMed, Embase, and the Web of Science until July 30, 2022. Chemotherapy, transcatheter arterial chemoembolization (TACE), molecular therapy (targeted therapy, immunotherapy, or a combination of both), and combined locoregional-systemic therapy were the conversion regimens we targeted.

**Results:**

Twenty-four studies were included. The pooled conversion rates for chemotherapy, TACE, molecular therapy, and combined locoregional-systemic therapy were 13% (95% confidence interval [CI], 7%–20%; I² = 82%), 12% (95% CI, 9%–15%; I² = 60%), 10% (95% CI, 3%–20%; I² = 90%), and 25% (95% CI, 13%–38%; I² = 89%), respectively. The pooled objective response rates (ORR) for chemotherapy, TACE, molecular therapy, and combined locoregional-systemic therapy were 19% (95% CI, 12%–28%; I² = 77%), 32% (95% CI, 15%–51%; I² = 88%), 30% (95% CI, 15%–46%; I² = 93%), and 60% (95% CI, 41%–77%; I² = 91%), respectively. The pooled grade ≥3 AEs for chemotherapy, TACE, molecular therapy, and combined locoregional-systemic therapy were 67% (95% CI, 55%–78%; I² = 79%), 34% (95% CI, 8%–66%; I²= 92%), 30% (95% CI, 18%–43%; I² = 84%), and 40% (95% CI, 23%–58%; I² = 89%), respectively. Subgroup analyses showed the conversion rate, ORR and grade ≥3 AE rate for tyrosine kinase inhibitor (TKI) combined with immune checkpoint inhibitor (ICI) and locoregional therapy (LRT) were 33% (95% CI, 17%-52%; I² = 89%), 73% (95% CI, 51%–91%; I² = 90%), 31% (95% CI, 10%-57%; I² = 89%), respectively.

**Conclusion:**

Combined locoregional-systemic therapy, especially TKI combined with ICI and LRT, may be the most effective conversion therapy regimen, associated with a significant ORR, conversion potential, and an acceptable safety profile.

## Introduction

Hepatocellular carcinoma (HCC) is the sixth most common malignant tumor in the world and ranks third in terms of the mortality rate of malignant tumors worldwide in 2020 (1). Apart from liver transplantation, which is limited by a lack of donors, hepatectomy is the only curative therapy that can achieve long-term survival for early HCC. Regrettably, >70% of individuals with HCC are diagnosed in a mid- or advanced stage due to the lack of symptoms in the early stages of the disease (2). As a result, these patients are considered unresectable and miss the window for radical hepatectomy (3, 4).

Current treatment options for intermediate and advanced HCC are non-surgical, such as locoregional therapy (LRT), and systemic therapy, which offer only poor long-term survival. Surprisingly, some selected patients with unresectable HCC (uHCC) have experienced tumor shrinkage and downstaging after LRT and systemic therapy, thus meeting the criteria for resectability (5, 6). This treatment strategy, which aims to convert uHCC into resectable HCC, is known as conversion therapy. Patients with uHCC who have undergone successful conversion and subsequent resection have a 5-year survival rate of >50% (7, 8), which is similar to the 5-year survival rate for patients with resectable HCC who have undergone surgical resection (9). The LRTs used for conversion therapy include transcatheter arterial chemoembolization (TACE), hepatic arterial infusion chemotherapy (HAIC), and transarterial radioembolization (TARE). The systemic treatments used for conversion therapy include chemotherapy, targeted therapy, and immunotherapy.

Recently, with the development and application of the new tyrosine kinase inhibitor (TKI) and immune checkpoint inhibitor (ICI), the efficacy of targeted therapies and immunotherapies for uHCC has improved compared to the past. Furthermore, the improved efficacy makes the targeted therapy and immunotherapy increasingly important in conversion therapy strategies for uHCC. On this basis, combinations of targeted therapies and immunotherapies, as well as combined locoregional-systemic therapy, have been used as conversion therapies. To date, a number of conversion therapy strategies have been investigated, but the best therapeutic treatment options remain unclear. Therefore, we conducted several meta-analyses to systematically evaluate the safety and efficacy of representative treatment strategies (chemotherapy, TACE, molecular therapy, and combined locoregional-systemic therapy) as conversion therapies for HCC in order to elucidate the optimal regimen.

## Methods

All items in our meta-analyses were reported according to the Preferred Reporting Items for Systematic Reviews and Meta-Analyses (PRISMA) statement (10).

### Search strategy

In these meta-analyses, relevant studies were systematically searched for in PubMed, Embase, and the Web of Science up to July 30, 2022. The search strings used were as follows: (“unresectable” OR “intermediate-stage” OR “advanced”) AND (“liver cancer” OR “hepatoma” OR “hepatic carcinoma” OR “hepatocellular carcinoma” OR “hepatocarcinoma”) AND (chemotherapy OR (“loco-regional therap*” OR “locoregional therap*”) OR (TACE OR “transcatheter arterial chemoembolization”) OR (“hepatic arterial infusion chemotherapy” OR HAIC) OR (radiotherapy OR (“Transarterial Radioembolization” OR TARE) OR yttrium-90 OR (“selective internal radiation therapy” OR SIRT) OR (“Stereotactic Body Radiation Therapy” OR SBRT)) OR (“Targeted therapy” OR “tyrosine kinase inhibitor*” OR “Immune checkpoint inhibitor*” OR “systemic therap*”) OR [(Triple therapy) OR (combination therapy) OR combined)] AND [(“hepatic resection” OR “liver resection” OR “hepatectomy”[Mesh]) OR resectable]. In addition, references listed in published articles that may be relevant to this review were manually searched.

### Literature selection

Included studies were required to meet the following criteria (1): enrolled patients who were initially diagnosed with potentially resectable uHCC (e.g., an Eastern Cooperative Oncology Group performance status [ECOG PS] score of 0–2 points and a Child–Pugh classification of A or B, despite the combination of extrahepatic metastases, macrovascular invasion [MVI], multiple tumors, or insufficient future liver remnant [FLR]); (2) the intervention included ≥1 of the treatments we studied (chemotherapy, TACE, molecular therapy, and combined locoregional-systemic therapy); (3) the outcomes included the conversion rate or the number of people successfully converted, the objective response rate (ORR), and the grade ≥ 3 treatment-related adverse events (AEs) rate; and (4) study types included randomized controlled trials (RCTs), non-RCTs, single-arm studies, cohort studies, case–control trials, or case series. Meanwhile, the exclusion criteria were as follows: (1) studies that included participants diagnosed with secondary liver cancer; (2) studies with mostly the same population (if multiple studies were found, the most recent or most detailed study was adopted); (3) incomplete or unavailable target outcome data; and (4) reviews, comments, conference, abstracts, letters, case reports, and animal experiments. Two authors independently browsed the titles and abstracts of all articles to identify articles relevant to our study. Finally, studies included in the meta-analysis were screened out by reading their full texts. Any disagreements were resolved through discussions with a third investigator.

### Data extraction

The primary outcome was the conversion rate, and the secondary outcomes were the ORR and grade ≥ 3 AE rate. The relevant data were extracted by two authors independently from the included studies and filled into a predesigned Excel sheet (Microsoft, Redmond, WA, USA). The data collected were as follows: (1) the first author, year of publication, country, study design, and the number of people receiving conversion therapy, and (2) conversion therapy modalities and schedule, conversion rate, ORR, grade ≥ 3 AE rate, reason of unresectability, and criteria of resectability. Any disagreements were resolved through discussions with a third investigator.

### Quality assessment

Because single-arm meta-analyses were used to quantify the pooled results, we used the methodological index for non-randomized studies (MINORS) tool (11) to assess the methodological quality of RCTs and non-RCTs as single-arm studies. Similarly, we used the Institute of Health Economics Quality Appraisal (IHEQA) Checklist (12) to assess the methodological quality of cohort and case–control studies as case series.

### Statistical analysis

Data analysis was performed using R version 4.1.2 (R Foundation for Statistical Computing, Vienna, Austria), and *P* < 0.05 indicated a statistically significant result. Heterogeneity was assessed using Cochran’s Q test and I² test, and I² > 50% or *P* < 0.1 indicated significant heterogeneity. When I² > 50%, a random-effects model was used; if I² ≤ 50%, a fixed-effects model was used. Then, the pooled event rate (conversion rate, ORR, and grade ≥ 3 AE rate) and 95% confidence interval (95% CI) were calculated using the “meta” package of R. In addition, funnel plots, and Egger’s tests were used to assess the publication biases.

## Results

### Study identification and characteristics

**Figure 1:**
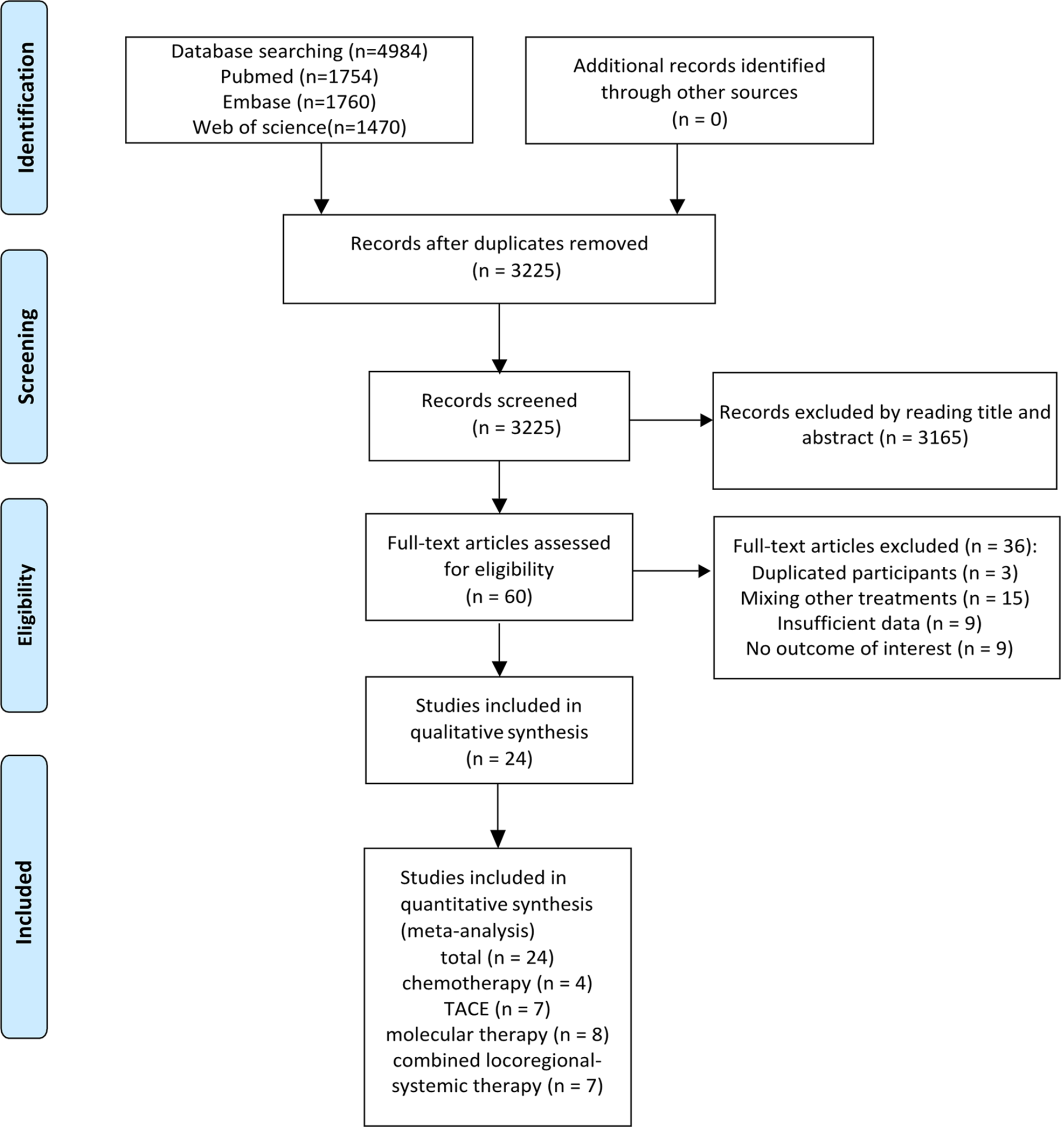
The flowchart for the study search and screening.

The initial search identified 4,984 references. A total of 3,225 records remained after removing duplicates, and 3,165 articles were further excluded by title and abstract screening. Subsequently, the remaining 60 articles were assessed for eligibility by reading their full texts, and 36 were further excluded (including three studies with duplicate participants, 15 studies with treatments mixed with other treatments, nine with insufficient data, and nine with no results of interest). Finally, 24 studies met the inclusion criteria and were included in these meta-analyses. **Figure 1** illustrates the flowchart for literature screening. The characteristics of the included studies are summarized in **Table 1**. In total, four studies were included in the chemotherapy group (7, 13–15), seven were included in the TACE group (8, 16–21), eight were included in the molecular therapy group (22–29), and seven were included in the combined locoregional-systemic therapy group (23, 24, 30–34). Nineteen studies (7, 8, 13, 15–17, 19–22, 24, 25, 27–29, 31–34) were considered to be of acceptable quality according to the IHEQA checklist, and the remaining five studies (14, 18, 23, 26, 30) were considered to be of moderate to high quality according to the MINORS tool. The details are summarized in the Supplementary Materials.

**Table 1:** Characteristics of included studies.

Study	Year	Group of interventions	Subgroup of interventions	N	Reason of unresectability	Definition of successful conversion	Design
Leung (13)	2002	CT	PIAF	149	Extrahepatic metastasis; MVI; Extensive disease	Downstaging to resectable	Case series
Lau-cohort 1 (7)	2004	CT	PIAF	128	Multiple tumors; MVI; Extensive bilobar involvement	Tumor shrinks and FLR increases to resectable	Case series
Yeo-cohort 1 (14)	2005	CT	PIAF	86	Extrahepatic metastasis	Downstaging to resectable	RCT
Kaseb-cohort 1 (15)	2013	CT	PIAF	84	Extrahepatic metastasis; MVI	Resectability was assessed by experienced hepatobiliary surgeons	Retrospective cohort
Kaseb-cohort 2 (15)	2013	CT	PIAF*	33	Extrahepatic metastasis; MVI	Resectability was assessed by experienced hepatobiliary surgeons	Retrospective cohort
Lau-cohort 2 (7)	2004	CT	Doxorubicin	76	Multiple tumors; MVI; Extensive bilobar involvement	Tumor shrinks and FLR increases to resectable	Case series
Yeo-cohort 2 (14)	2005	CT	Doxorubicin	94	Extrahepatic metastasis;	Downstaging to resectable	RCT
Fan (8)	1998	TACE	cTACE	360	Insufficient FLR; Oversized tumors	Tumor shrinks to resectable	Case series
Shi (16)	2012	TACE	cTACE	420	Insufficient FLR; Oversized tumors	Tumor shrinks to resectable	Case series
Zhang (17)	2016	TACE	cTACE	831	Multiple tumors; Insufficient FLR;	R0 resection	Retrospective cohort
He (18)	2017	TACE	cTACE	41	Oversized tumors	Tumor shrinks to resectable	nRCT
Wu-cohort 1 (19)	2018	TACE	cTACE	30	BCLC stage B/C	Downstaging to resectable	Retrospective cohort
Chiu-cohort 1 (20)	2020	TACE	cTACE	19	MVI	Downstaging to resectable	Retrospective cohort
Li (21)	2021	TACE	cTACE	42	Insufficient FLR	Adequate FLR	Retrospective cohort
Wu-cohort 2 (19)	2018	TACE	DEB-TACE	24	BCLC stage B/C	Downstaging to resectable	Retrospective cohort
Chiu-cohort 2 (20)	2020	TACE	DEB-TACE	42	MVI	Downstaging to resectable	Retrospective cohort
Yoshimoto (22)	2018	MT	TKI	38	Advanced HCC	Tumor shrinks to resectable	Case series
He-cohort 1 (23)	2019	MT	TKI	122	MVI	Downstaging to resectable	RCT
He-cohort 1 (24)	2021	MT	TKI	86	Advanced HCC; BCLC stage C	Tumor shrinks to resectable	Retrospective cohort
Shindoh (25)	2021	MT	TKI	107	Advanced HCC	R0 resection	Case series
Zhang (26)	2020	MT	TKI+ICI	33	MVI	Adequate FLR	Prospective single-arm
Zhu (27)	2021	MT	TKI+ICI	63	Mid- or advanced HCC; Insufficient FLR	R0 resection with adequate FLR; Good physical condition	Case series
Huang (28)	2021	MT	TKI+ICI	60	Extrahepatic metastases; MVI	Downstaging to resectable	Case series
Xie (29)	2021	MT	TKI+ICI	60	Confirmed histologically or radiologically	Downstaging to resectable with adequate FLR	Case series
He (30)	2018	LRT+systemic treatment	TKI+LRT	35	MVI	Downstaging to resectable	Prospective single-arm
He-cohort 2 (23)	2019	LRT+systemic treatment	TKI+LRT	125	MVI	Downstaging to resectable	RCT
Chen-cohort 1 (31)	2021	LRT+systemic treatment	TKI+LRT	72	Mid- or advanced-stage HCC; Insufficient FLR	Downstaging to resectable	Retrospective cohort
He-cohort 2 (24)	2021	LRT+systemic treatment	TKI+ICI+LRT	71	Advanced HCC; BCLC stage C	Tumor shrinks to resectable	Retrospective cohort
Yang (32)	2021	LRT+systemic treatment	TKI+ICI+LRT	38	Technical and/or oncological reasons	Downstaging to resectable	Case series
Zhang (33)	2021	LRT+systemic treatment	TKI+ICI+LRT	25	BCLC stage C	Adequate FLR	Case series
Wu (34)	2021	LRT+systemic treatment	TKI+ICI+LRT	62	Extensive bilobar involvement; MVI; Extrahepatic metastases	R0 resection with adequate FLR; Good physical condition	Case series
Chen-cohort 2 (31)	2021	LRT+systemic treatment	TKI+ICI+LRT	70	Mid- or advanced-stage HCC; Insufficient FLR	Downstaging to resectable	Retrospective cohort

N, number of patients with unresectable hepatocellular carcinoma; CT, chemotherapy; MT, Molecular therapy; LRT, locoregional therapy; PIAF, Cisplatin, interferon α-2b, 5-fluorouracil and doxorubicin; MVI, Macrovascular invasion; TACE, transcatheter arterial chemoembolization; cTACE, conventional transcatheter arterial chemoembolization; DEB-TACE, drug-eluding beads transcatheter arterial chemoembolization, TKI, Tyrosine kinase inhibitor; ICI, immune checkpoint inhibitor; HCC, hepatocellular carcinoma; BCLC, Barcelona Clinic Liver Cancer; FLR, future liver remnant;

*Modified PIAF.

### Chemotherapy

Four studies (7, 13–15), including seven subgroups, reported that the treatment modality was chemotherapy. The conversion rate, ORR, and the rate of grade ≥ 3 AEs were reported in seven subgroups of all studies, five subgroups of three studies (13–15), and four subgroups of two studies (14, 15), respectively. All studies included a total of 650 patients with uHCC. Most participants had extrahepatic metastases, vascular invasion, or multiple tumors. The Child–Pugh classification was mostly class A, and the EOCG PS was mostly 0–1 points. When focusing on treatment alternatives, all studies utilized a combination chemotherapy regimen (i.e., PIAF, cisplatin, interferon-2b, doxorubicin, and 5-fluorouracil), and two studies (7, 14) chose a single-agent doxorubicin chemotherapy regimen. The year of publication of the included studies ranged from 2002 to 2013.

**Figure 2:**
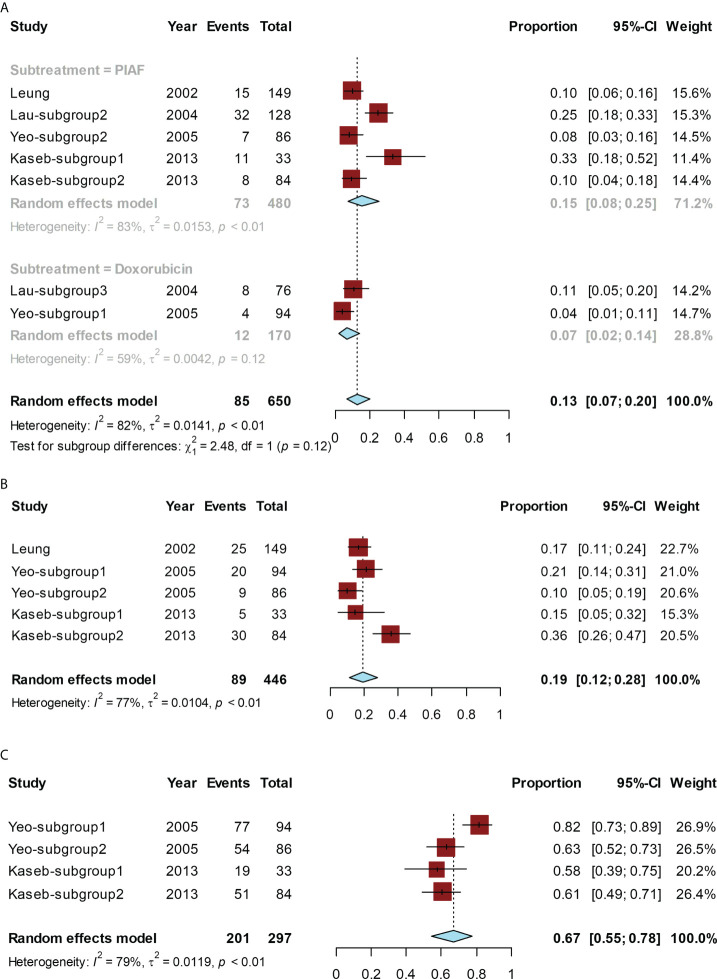
Forest plot for the chemotherapy group. The pooled conversion rate and subgroup analysis of the conversion rate according to PIAF or doxorubicin **(A)**, pooled ORR **(B)**, and the pooled rate of grade ≥ 3 AEs **(C)**.

The conversion rate for all studies ranged from 4% (14) to 33% (15). The pooled conversion rate was 13% (95% CI, 7%–20%; I² = 82%). The conversion rate of PIAF was 15% (95% CI, 8%–25%; I² = 83%) and that of doxorubicin alone was 7% (95% CI, 2%–14%; I² = 59%). The conversion rate of PIAF showed a non-significant trend of improvement compared to that of doxorubicin (*P* = 0.12) (**Figure 2A**).

The ORR ranged from 10% (14) to 36% (15), and the pooled ORR was 19% (95% CI, 12%–28%; I² = 77%) (**Figure 2B**).

The pooled rate of grade ≥ 3 AEs ranged from 58% (15) to 82% (14), and the pooled rate was 67% (95% CI, 55%–78%; I² = 79%) (**Figure 2C**).

### TACE

TACE was reported as an intervention in seven studies (8, 16–21) covering nine subgroups. Of these, nine subgroups of all studies reported conversion rates, six subgroups of four studies (18–21) reported ORRs, and three subgroups of two studies (18, 20) reported AEs of grade ≥ 3. In all studies, among 1,809 patients diagnosed with uHCC, the majority of participants had no extrahepatic metastases or MVI. In addition, most were classified as Child–Pugh class A and had an ECOG PS of 0–1 points. Considering anti-neoplastic drugs, all studies except Fan et al. (8) used doxorubicin or epirubicin. A few studies also used platinum, mitomycin (8, 18, 21), and 5-fluorouracil (8). Lipiodol or gelatin sponge was used in seven subgroups of all studies (conventional TACE [c-TACE]) to embolize target vessels, and drug-eluting beads (drug-eluting beads TACE [DEB-TACE]) were used in two subgroups of two studies (19, 20). The year of study publication ranged from 2012 to 2021, except for that by Fan et al. (8), which was published in 1998.

**Figure 3:**
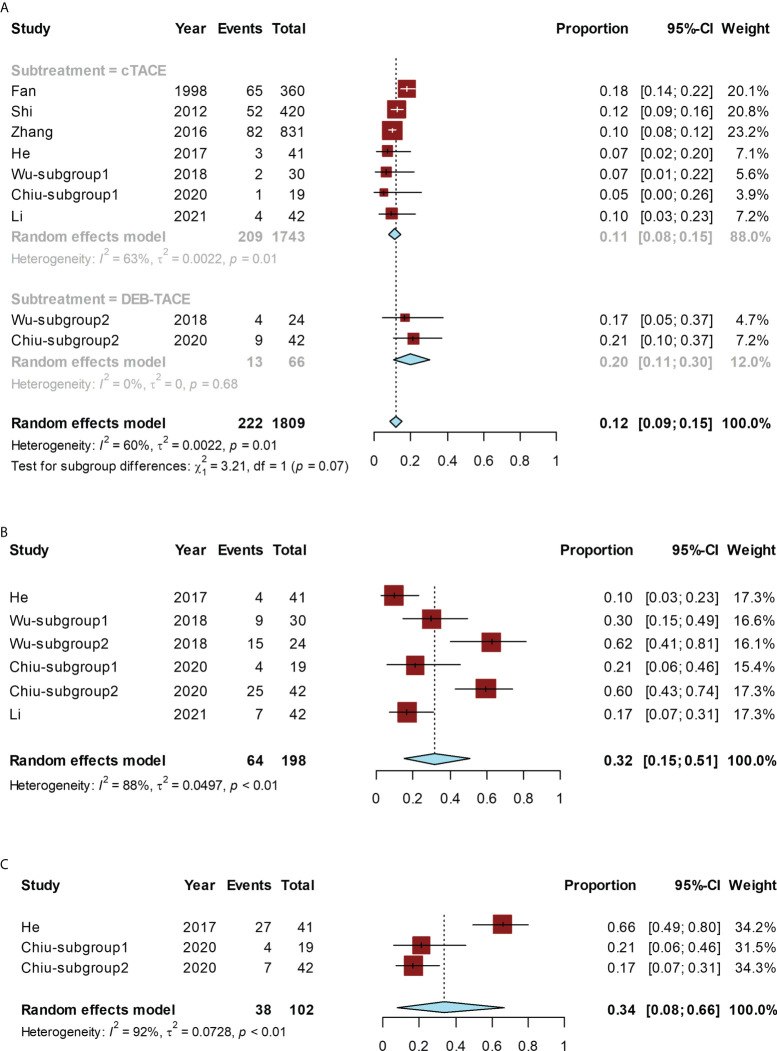
Forest plot for the TACE group. The pooled conversion rate and subgroup analysis of the conversion rate according to cTACE or DEB-TACE **(A)**, pooled ORR **(B)**, or pooled rate of grade ≥ 3 AEs **(C)**. cTACE, conventional transcatheter arterial chemoembolization; DEB-TACE, drug-eluding beads transarterial chemoembolization.

The conversion rate for all studies ranged from 5% (20) to 21% (20). The pooled conversion rate was 12% (95% CI, 9%–15%; I² = 60%). Subgroup analysis was performed depending on c-TACE/DEB-TACE. In the cTACE group, the conversion rate was 11% (95% CI, 8%–15%; I² = 63%), while, in the DEB-TACE group, the conversion rate was 20% (95% CI, 11%–30%; I² = 0). DEB-TACE had a higher conversion rate than c-TACE, but the difference was not statistically significant (*P* = 0.07) (**Figure 3A**).

The ORR ranged from 10% (18) to 62% (19), and the pooled ORR was 32% (95% CI, 15%–51%; I² = 88%) (**Figure 3B**).

The rate of grade ≥ 3 AEs ranged from 17% (20) to 66% (18), and the pooled rate was 34% (95% CI, 8%–66%), with significant heterogeneity (I² = 92%) (**Figure 3C**).

### Molecular therapy

There were eight studies (22–29), including eight subgroups, which adopted molecular therapy as the arm-treatment. All eight subgroups of all studies reported the conversion rate, six subgroups of six studies (23–26, 28, 29) reported ORR, and four subgroups of four studies (23, 25, 28, 29) reported AEs of grade ≥ 3. A total of 569 patients with uHCC were enrolled in all trials. Most participants were diagnosed with extrahepatic metastases, MVI, or multiple tumors. Meanwhile, almost all of them were classified as Child–Pugh class A and had an ECOG PS of 0–1 points. Four studies (22–25) adopted TKI alone, and four studies (26–29) adopted TKI combined with ICI. The TKIs used in most studies were sorafenib (22, 23) and lenvatinib (24–29), with only one study using apatinib (27). The ICIs were various anti–programmed cell death protein 1 antibodies (e.g., sindilizumab, pabrolizumab, camrelizumab, and toripalimab). The years of study publication ranged from 2018 to 2021.

**Figure 4:**
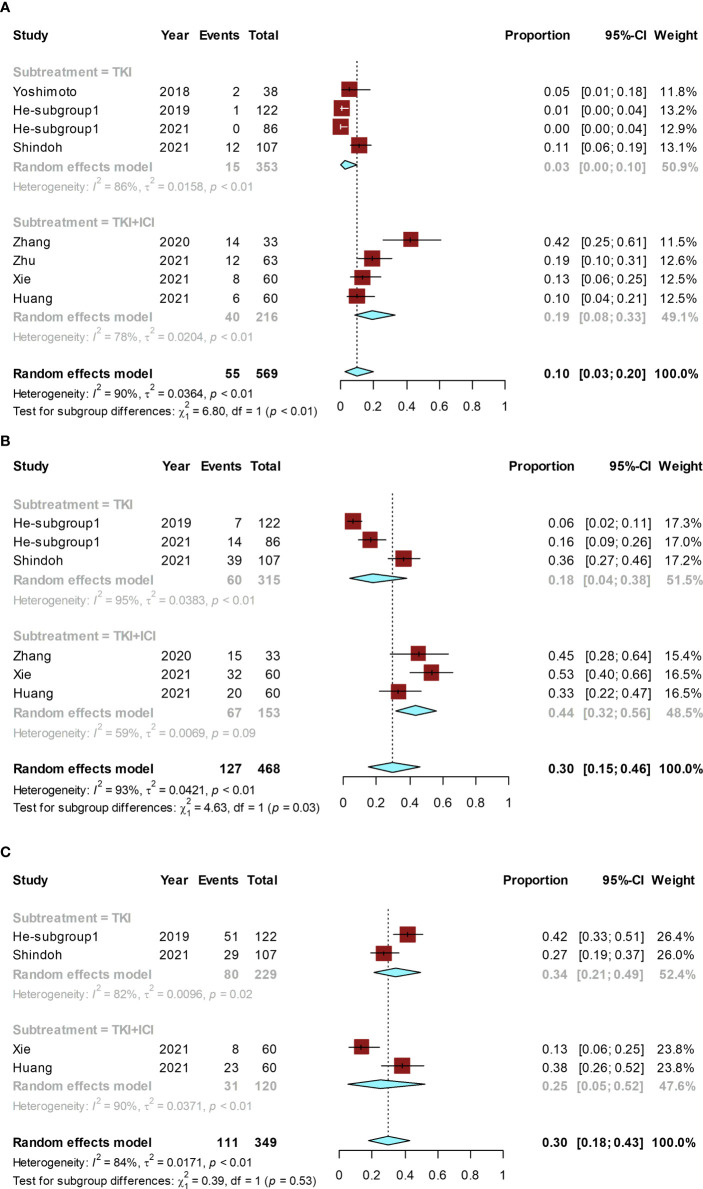
Forest plot for the molecular therapy group. Pooled rates and the subgroup analysis of conversion rate according to the use of TKI alone or TKI combined with ICI: pooled conversion rate **(A)**, pooled ORR **(B)**, and the pooled rate of grade ≥ 3 AEs **(C)**. TKI, tyrosine kinase inhibitor; ICI, immune checkpoint inhibitor.

The conversion rate of included studies ranged from 0% (24) to 42% (26), and the pooled conversion rate was 10% (95% CI, 3%–20%; I² = 90%). A subgroup analysis was performed based on monotherapy with TKI alone or TKI combined with ICI. The conversion rate was 19% (95% CI, 8%–33%; I² = 78%) in the group receiving TKI combined with ICI and 3% (95% CI, 0–10%; I² = 86%) in the TKI-alone group. The conversion rate in the group receiving TKI combined with ICI was significantly higher than that in the TKI-alone group (*P* < 0.01) (**Figure 4A**).

The ORR ranged from 6% (23) to 53% (29) and the pooled ORR was 30% (95% CI, 15%–46%; I² = 93%). The ORR was 44% (95% CI, 32%–56%; I² = 59%) in TKI combined with ICI group and 18% (95% CI, 4%–38%; I² = 95%) in the TKI-alone group. The ORR of TKI combined with ICI was significantly higher than that of the TKI-alone (*P* = 0.03) (**Figure 4B**).

The grade ≥ 3 AE rate ranged from 13% (29) to 42% (23), and the pooled rate was 30% (95% CI, 18%–43%; I² = 84%). The grade ≥ 3 AE rate was 25% (95% CI, 5%–52%; I² = 90%) in TKI combined with ICI group and 34% (95% CI, 21%–49%; I² = 82%) in the TKI-alone group. No significant difference existed in the grade ≥ 3 AE rate between TKI combined with ICI group and the TKI-alone group (*P* = 0.53) (**Figure 4C**).

### Combined locoregional-systemic therapy

Eight subgroups in seven studies (23, 24, 30–34) reported combined locoregional-systemic therapy. The conversion rates and ORR were available for eight subgroups and seven subgroups from all studies, respectively, and five subgroups from five studies (23, 30, 32–34) investigated the rates of grade ≥ 3 AEs. There were 498 patients with uHCC in all the studies. Most patients had the following baseline characteristics: concurrent extrahepatic metastases, MVI, or multiple tumors; Child–Pugh class A; Barcelona Clinic Liver Cancer (BCLC) stage C; and ECOG PS 0–1 points. For treatment strategies, five studies (24, 31–34) adopted TKI combined with ICI and LRT, and three studies (23, 30, 31) adopted TKI combined with LRT. The TKI used was lenvatinib (24, 31–34) or sorafenib (23, 30), and the ICI were various programmed cell death protein 1 monoclonal antibodies. For TACE, two studies (32, 34) used c-TACE, and one study (31) used DEB-TACE. For HAIC, all studies used the FOLFOX regimen. The years of study publication ranged from 2018 to 2021.

**Figure 5:**
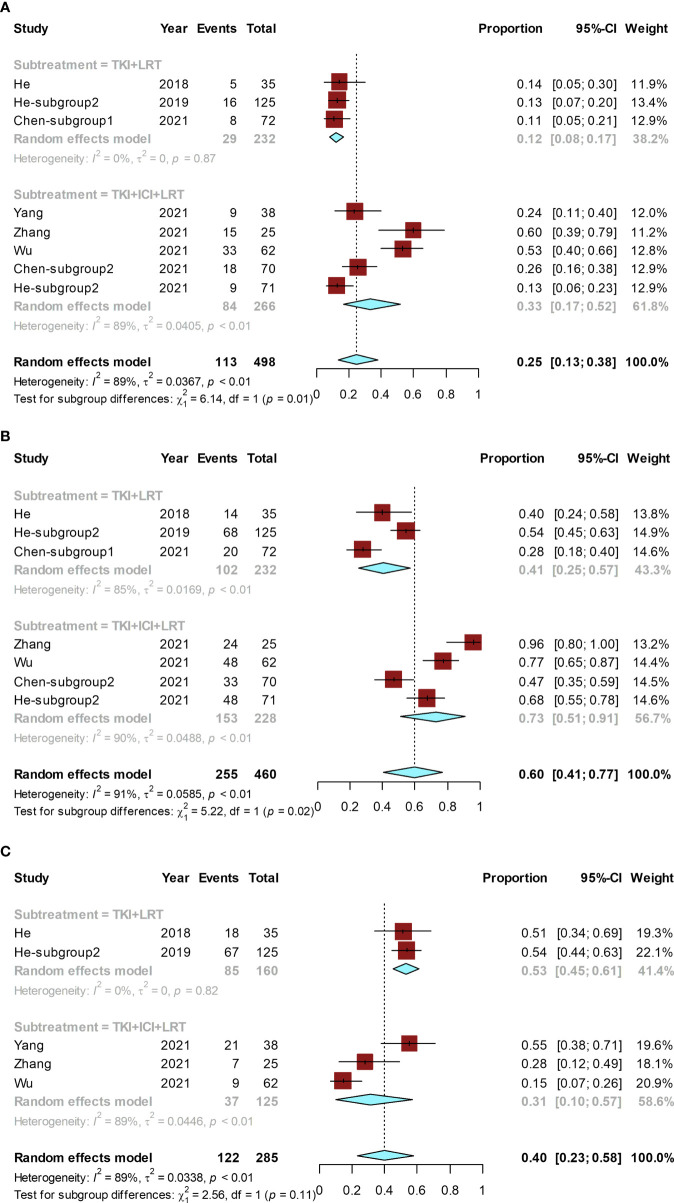
Forest plot for the combined locoregional-systemic therapy group. The pooled conversion rate and subgroup analysis **(A)**, pooled ORR and its subgroup analysis **(B)**, and the pooled rate of grade ≥ 3 AEs and its subgroup **(C)**. These subgroup analyses were conducted according to combination of treatments. LRT, locoregional therapy.

The conversion rates of available studies ranged from 11% (31) to 60% (33), and the pooled rate was 25% (95% CI, 13%–38%; I² = 89%). A subgroup analysis was performed according to the combination of treatments. The pooled conversion rate for the TKI combined with ICI and LRT was 33% (95% CI, 17%-52%; I² = 89%), which was significantly higher than that for TKI combined with LRT (12% [95% CI, 8%-17%; I² = 0%]) (P = 0.01) (**Figure 5A**).

The ORR of included studies ranged from 28% (31) to 96% (33), and the pooled ORR was 60% (95% CI, 41%–77%; I² = 91%). Subgroup analysis suggested that the pooled ORR of TKI combined with ICI and LRT was 73% (95% CI, 51%–91%; I² = 90%), while the pooled ORR of TKI combined with LRT was 41% (95% CI, 25%–57%; I² = 85%) (**Figure 5B**). The ORR of TKI combined with ICI and LRT was significantly higher than that of TKI combined with LRT (P = 0.02).

The grade ≥ 3 AE rate of included studies ranged from 15% (34) to 55% (32), and the pooled grade ≥ 3 AE rate was 40% (95% CI, 23%–58%; I² = 89%) (**Figure 5C**). The grade ≥ 3 AE rate between the TKI combined with ICI and LRT group (31% [95% CI, 10%-57%; I² = 89%]) and the TKI combined with LRT group (53% [95% CI, 45%-61%; I² = 0%]) was not statistically significantly different (P = 0.11).

### Publication bias

**Figure 6:**
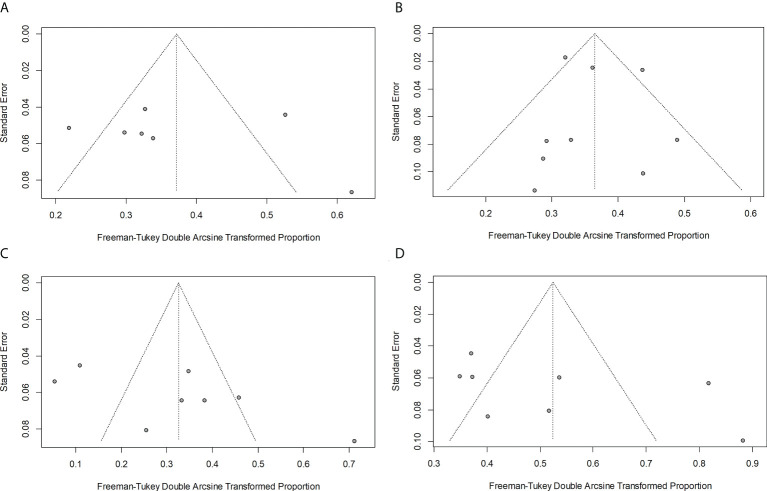
Funnel plots for the conversion rates of chemotherapy **(A)**, TACE **(B)**, molecular therapy **(C)**, and combined locoregional-systemic therapy **(D)**.

No significant publication bias existed according to the funnel plots (**Figure 6**) and Egger’s test (**Supplementary Figure S1**) based on an analysis of the conversion rate of chemotherapy (*P* = 0.625), TACE (*P* = 0.776), molecular therapy (*P* = 0.087), and combined locoregional-systemic therapy (*P* = 0.190) groups.

## Discussion

With the advent and development of new biologic agents and the exploration of treatment strategies, uHCC, once considered incurable, can become resectable with conversion therapy and achieve survival benefits comparable to those achieved with resection of early-stage HCC (7–9). There are many options for conversion therapy, but the best choice is not yet clear.

Our meta-analyses summarized the efficacy and safety of four representative types of conversion therapy for uHCC. Among these, chemotherapy, TACE, and molecular therapies had lower and similar conversion rates, whereas combined locoregional-systemic therapy had a significantly higher conversion rate. Notably, subgroup analysis showed no significant differences in conversion potential between different strategies of the same monotherapy. However, the conversion rate of the combined therapy was significantly better than that of the monotherapy. The increased conversion potential of combined therapy could be since the fact that different treatments have different anti-tumor mechanisms. In particular, TKI combined with ICI and LRT has the highest conversion rate (33%) compared to any other treatment strategy, which is close to the 39.1% rate of conversion surgery for FOLFOXIRI plus bevacizumab as a conversion therapy used for patients with initially unresectable metastatic colorectal cancer (35), which is exciting.

The ORRs achieved with chemotherapy, TACE, and molecular therapy remained similar. Similarly, the ORR for combined locoregional-systemic therapy remained significantly higher than the ORRs of the aforementioned other therapies. Similar to the trend in the subgroup analysis of the conversion rate, combined therapy was associated with a higher ORR, and TKI combined with ICI and LRT could achieve the highest ORR. To some extent, this result suggested that strategies that can have a higher ORR may imply a higher conversion potential.

In terms of safety, we were mainly concerned about serious (grade ≥ 3) treatment-related AEs. The chemotherapy group had the worst safety profile, with around 70% of patients experiencing significant side effects. Given the low ORR and conversion rates of chemotherapy, its poor safety profile seems unacceptable today. Safety was similar and acceptable in both the TACE group, the molecular therapy group, and the combined locoregional-systemic therapy group. Interestingly, the subgroup analysis showed increased safety risks with combination therapies compared to monotherapy, but the trend was insignificant. For the combined therapy, the safety of TKI combined with ICI was comparable to that of TKI combined with LRT. Furthermore, no increased security risks were identified even when comparing TKI combined with ICI and LRT with TKI combined with LRT.

Our findings additionally reflect the history and development of conversion therapy for uHCC to some extent. In the early stages, the options used as conversion therapy were mainly chemotherapy and LRT, represented by TACE. For chemotherapy, there are combination chemotherapy regimens (such as PIAF) and single-agent chemotherapy regimens (such as doxorubicin). Chemotherapy is currently rarely considered as conversion therapy for HCC due to its low conversion potential and high safety risks. However, LRT is continuing to develop. Representative TACE is currently used as the first-line treatment for intermediate to advanced HCC (36–38). In recent years, a new TACE approach (DEB-TACE) has been developed with the ability to increase the intravascular drug concentration and reduce the amount of chemotherapeutic drugs entering the systemic circulation (39). This ability might be why DEB-TACE was associated with greater conversion and improved safety compared to cTACE, although the difference was not statistically significant. Several studies (40–42) has shown that TARE could lead to tumor shrinkage and downstaging. However, due to liver resection mixed with liver transplantation following tumor downstaging, the role of TARE as conversion therapy for uHCC could not be accurately clarified.

Sorafenib was approved by the U.S. Food and Drug Administration for advanced uHCC in 2007. Sorafenib application extends the median survival time for patients with uHCC (43). However, the ORR of the included studies with sorafenib as the conversion therapy was only 6%, which implies a very low conversion potential (2%) (22, 23). Recently, significant progress has been made in developing new anti-tumor molecular drugs, including other TKIs and ICIs. Although the efficacy of single agents remains limited, TKI combined with ICI significantly improved the conversion rate but was accompanied by an increased incidence of AEs. The inference that drugs with different anti-tumor mechanisms have increased conversion potential when used in combination seems reasonable. It might have been based on this inference that the combination of LRT and systemic therapy has recently received more attention, with higher conversion rates as expected. In particular, triple therapy consisting of TKIs combined with ICIs plus an LRT began to be extensively studied in 2021, with a higher conversion rate than any other.

Admittedly, some limitations should be pointed out. First, a high degree of heterogeneity exists in this meta-analysis. Its sources may be as follows (1): differentiation of unresectable causes and inconsistent criteria for resectability, and (2) there are no fixed criteria for the choice of treatment regimen and drug dose. So, subgroup analysis was performed to explore the stability of the results and further interpret the results. Second, most included studies were not using conversion rates as the primary endpoint since conversion therapy for HCC has only recently received attention. In addition, the population characteristics of the groups were inconsistent. All of our studies included patients with extrahepatic metastases, except for the TACE group, which did not include patients with extrahepatic metastases. The inconsistency in population characteristics might be primarily due to the different indications for different treatment strategies. So, our study focused on each treatment strategy.

The exploration of transformation therapy for uHCC is in the ascendant. Prospective controlled trials with large samples of different combinations of conversion strategies should be performed more often to provide better-quality evidence for clinical practice. Following conversion therapy strategies, criteria for resectability and study endpoints have yet to be further harmonized for uHCC. In the future, individualized protocols and studies for conversion therapy may receive more attention due to the biological heterogeneity of primary HCC.

## Conclusion

Our findings demonstrated that combined locoregional-systemic therapy, may be the most effective conversion therapy regimen for uHCC at present, which is associated with a significant ORR and conversion potential, along with an acceptable safety profile.

## Author contributions

JL and YP contributed to the conception and design of the study. YP and WL conducted the literature search and extracted the data. ZW was involved in the resolution of all the arguments. YP conducted the data analysis and wrote the manuscript. All authors contributed to the article and approved the submitted version.

## Funding

This research was supported by the Hebei Provincial Key Research and Development Program Project (21377767D) and the Hebei Provincial Postgraduate Innovation Fund (CXZZSS2022137).

## Acknowledgments

We thank LetPub (www.letpub.com) for its linguistic assistance during the preparation of this manuscript.

## Conflict of interest

The authors declare that the research was conducted in the absence of any commercial or financial relationships that could be construed as a potential conflict of interest.

## Publisher’s note

All claims expressed in this article are solely those of the authors and do not necessarily represent those of their affiliated organizations, or those of the publisher, the editors and the reviewers. Any product that may be evaluated in this article, or claim that may be made by its manufacturer, is not guaranteed or endorsed by the publisher.
