# Supplementary material for: Successful conversion therapy for unresectable hepatocellular carcinoma is getting closer: A systematic review and meta-analysis
Source: Front Oncol. 2022 Sep 13;12:978823. doi: 10.3389/fonc.2022.978823 (PMC9513549; doi:10.3389/fonc.2022.978823)
Supplement: Supplementary file 1 [file DataSheet_1.docx]

**Supplemental Table 1 Methodological quality assessment for cohort studies and case series included in the meta-analysis using the IHEQA tool**

| Criteria | Leung 2002 | Lau 2004 | Kaseb 2013 | Fan 1998 | Shi 2012 | Zhang 2016 | Wu 2018 | Yoshimoto 2018 | Chiu 2020 | Li 2021 | He 2021 | Chen 2021 | Zhu 2021 | Xie 2021 | Huang 2021 | Shindoh 2021 | Yang 2021 | Zhang 2021 | Wu 2021 |
| --- | --- | --- | --- | --- | --- | --- | --- | --- | --- | --- | --- | --- | --- | --- | --- | --- | --- | --- | --- |
| **﻿Study objective** |  |  |  |  |  |  |  |  |  |  |  |  |  |  |  |  |  |  |  |
| ﻿1. A | Y | Y | Y | Y | Y | Y | Y | Y | Y | Y | Y | Y | Y | Y | Y | Y | Y | Y | Y |
| **Study design** |  |  |  |  |  |  |  |  |  |  |  |  |  |  |  |  |  |  |  |
| ﻿2. B | N | N | N | N | N | N | N | N | N | N | N | N | N | N | N | N | N | N | N |
| ﻿3. C | N | N | N | N | U | N | N | N | N | U | Y | Y | U | N | N | N | N | N | Y |
| ﻿4. D | U | Y | U | U | U | Y | U | U | U | U | Y | Y | Y | U | U | Y | U | U | U |
| **﻿Study population** |  |  |  |  |  |  |  |  |  |  |  |  |  |  |  |  |  |  |  |
| ﻿5. E | Y | Y | Y | Y | Y | Y | Y | Y | Y | Y | Y | Y | Y | Y | Y | Y | Y | Y | Y |
| ﻿6. F | Y | Y | P | Y | N | Y | Y | P | Y | Y | Y | Y | P | Y | P | Y | P | Y | Y |
| ﻿7. G | Y | Y | Y | Y | Y | Y | Y | Y | Y | Y | Y | Y | Y | Y | Y | Y | Y | Y | Y |
| **﻿Intervention and co-intervention** |  |  |  |  |  |  |  |  |  |  |  |  |  |  |  |  |  |  |  |
| ﻿8. H | Y | Y | Y | Y | Y | Y | Y | Y | Y | Y | Y | Y | Y | Y | Y | Y | Y | Y | Y |
| ﻿9. I | Y | Y | Y | Y | Y | Y | Y | Y | Y | Y | Y | Y | Y | Y | Y | Y | Y | Y | Y |
| **﻿Outcome measure** |  |  |  |  |  |  |  |  |  |  |  |  |  |  |  |  |  |  |  |
| ﻿10. J | Y | Y | Y | N | N | Y | Y | Y | Y | Y | Y | Y | Y | Y | Y | Y | Y | Y | Y |
| ﻿11. K | U | U | U | U | U | U | U | U | U | U | U | U | U | U | U | U | U | U | U |
| ﻿12. L | Y | Y | Y | Y | Y | Y | Y | Y | Y | Y | Y | Y | Y | Y | Y | Y | Y | Y | Y |
| ﻿13. M | Y | N | Y | N | Y | Y | Y | Y | Y | Y | Y | Y | Y | Y | Y | Y | Y | Y | Y |
| **﻿Statistical analysis** |  |  |  |  |  |  |  |  |  |  |  |  |  |  |  |  |  |  |  |
| ﻿14. O | Y | Y | Y | Y | Y | Y | Y | Y | Y | Y | Y | Y | Y | Y | Y | Y | Y | Y | Y |
| **﻿Results and conclusions** |  |  |  |  |  |  |  |  |  |  |  |  |  |  |  |  |  |  |  |
| ﻿15. Q | N | Y | Y | N | Y | Y | Y | N | Y | Y | Y | Y | Y | Y | Y | Y | Y | Y | Y |
| ﻿16. R | N | N | Y | N | N | N | Y | N | Y | N | N | N | N | N | N | N | N | N | N |
| ﻿17. S | Y | Y | N | N | N | N | N | N | N | N | N | N | N | N | N | N | N | Y | N |
| ﻿18. T | N | Y | Y | N | N | Y | Y | N | Y | Y | Y | Y | Y | Y | Y | N | Y | Y | Y |
| ﻿19. V | Y | Y | Y | Y | Y | Y | Y | Y | Y | Y | Y | Y | Y | Y | Y | Y | Y | Y | Y |
| **﻿Competing interests and sources of support** |  |  |  |  |  |  |  |  |  |  |  |  |  |  |  |  |  |  |  |
| ﻿20. W | N | N | N | N | N | N | N | N | N | N | N | N | N | N | N | N | N | N | N |

A, Was the hypothesis/aim/objective of the study clearly stated?

B, Was the study conducted prospectively?

C, Were the cases collected in more than one centre?

D, Were patients recruited consecutively?

E, Were the characteristics of the patients included in the study described?

F, Were the eligibility criteria (i.e. inclusion and exclusion criteria) for entry into the study clearly stated?;

G, Did patients enter the study at a similar point in the disease?;

H, Was the intervention of interest clearly described?;

I, Were additional interventions (co-interventions) clearly described?;

J, Were relevant outcome measures established a priori?

K, Were outcome assessors blinded to the intervention that patients received?;

L, Were the relevant outcomes measured using appropriate objective/subjective methods?

M, Were the relevant outcome measures made before and after the intervention?;

O, Were the statistical tests used to assess the relevant outcomes appropriate?;

Q, Was follow-up long enough for important events and outcomes to occur?

R, Were losses to follow-up reported?

S, Did the study provided estimates of random variability in the data analysis of relevant outcomes?

T, Were the adverse events reported?

V, Were the conclusions of the study supported by results?

W, Were both competing interests and sources of support for the study reported?

Y, yes; N, no; N/A, not applicable; U, unclear from study report; P, partially reported.

**Supplementary Table 2 Methodological quality assessment for RCT, nRCT, and**

**single-arm studies included in the meta-analysis using the MINORS tool**

| Study | A clearly stated aim | Inclusion of consecutive patients | Prospective collection of data | Endpoints appropriate to the aim of the study | Unbiased assessment of the study endpoint | Follow-up period appropriate to the aim of the study | Loss to follow up less than 5% | Prospective calculation of the study size | Total | Quality |
| --- | --- | --- | --- | --- | --- | --- | --- | --- | --- | --- |
| He 2017 | 2 | 2 | 2 | 2 | 2 | 2 | 1 | 0 | 13 | High |
| He  2017 | 2 | 2 | 2 | 2 | 2 | 2 | 1 | 0 | 13 | High |
| Zhang 2020 | 2 | 2 | 1 | 2 | 2 | 1 | 1 | 0 | 11 | Median |
| Yeo 2005 | 2 | 2 | 2 | 2 | 2 | 2 | 1 | 0 | 13 | High |
| He 2019 | 2 | 2 | 2 | 2 | 2 | 2 | 2 | 0 | 14 | High |
|  |  |  |  |  |  |  |  |  |  |  |

**
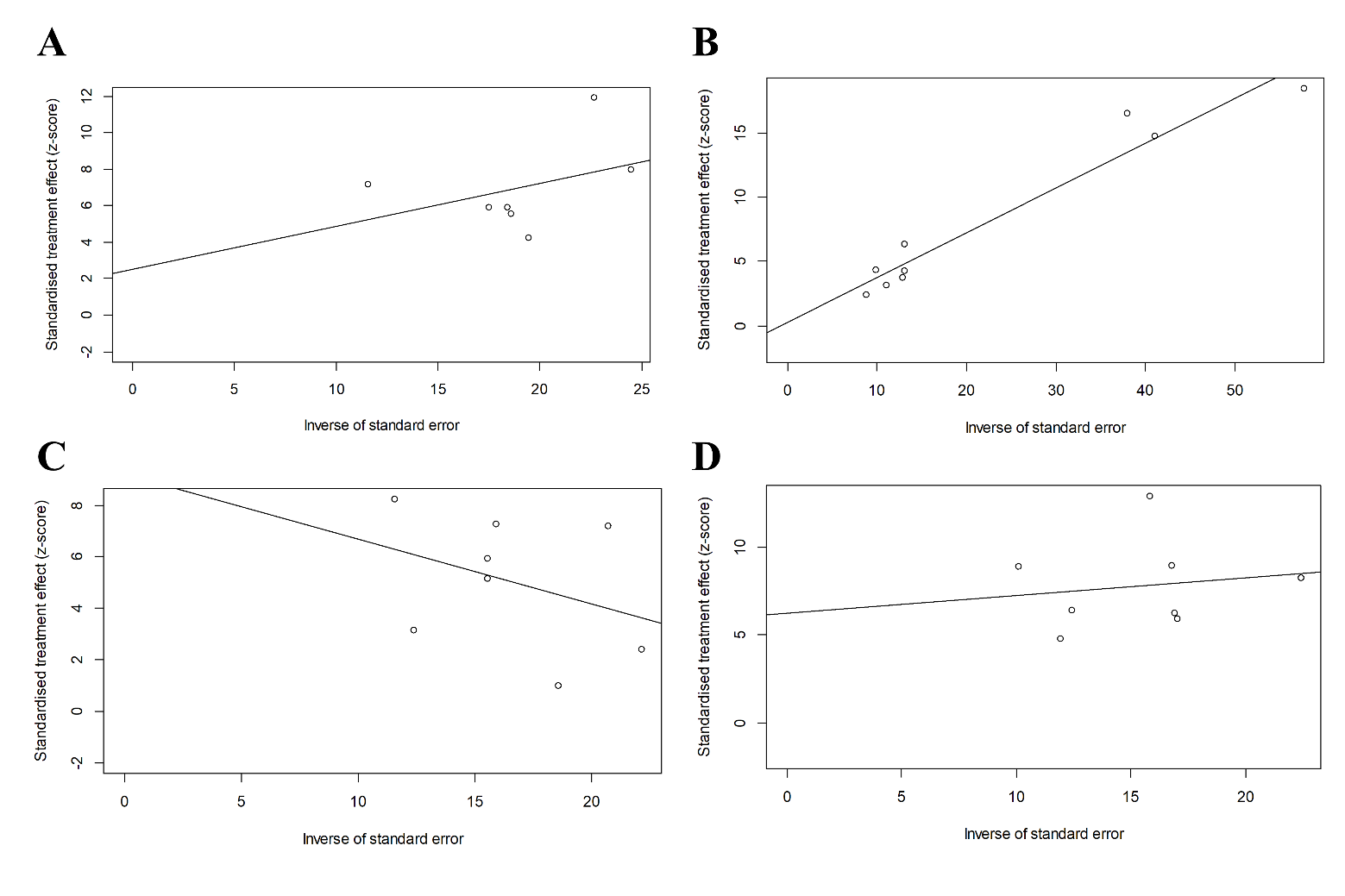
**

**Supplementary FIGURE 1.** Results of Egger’s test for the conversion rates of chemotherapy (**A**), TACE (**B**), molecular therapy (**C**), and combined locoregional-systemic therapy (**D**).
